# Neurofibromatosis type 2 (NF2): A clinical and molecular review

**DOI:** 10.1186/1750-1172-4-16

**Published:** 2009-06-19

**Authors:** D Gareth R Evans

**Affiliations:** 1 Medical Genetics Research Group, Regional Genetics Service and National Molecular Genetics Reference Laboratory, Central Manchester Foundation Trust, St Mary's Hospital, Manchester M13 0JH, UK

## Abstract

Neurofibromatosis type 2 (NF2) is a tumour-prone disorder characterised by the development of multiple schwannomas and meningiomas. Prevalence (initially estimated at 1: 200,000) is around 1 in 60,000. Affected individuals inevitably develop schwannomas, typically affecting both vestibular nerves and leading to hearing loss and deafness. The majority of patients present with hearing loss, which is usually unilateral at onset and may be accompanied or preceded by tinnitus. Vestibular schwannomas may also cause dizziness or imbalance as a first symptom. Nausea, vomiting or true vertigo are rare symptoms, except in late-stage disease. The other main tumours are schwannomas of the other cranial, spinal and peripheral nerves; meningiomas both intracranial (including optic nerve meningiomas) and intraspinal, and some low-grade central nervous system malignancies (ependymomas). Ophthalmic features are also prominent and include reduced visual acuity and cataract. About 70% of NF2 patients have skin tumours (intracutaneous plaque-like lesions or more deep-seated subcutaneous nodular tumours). Neurofibromatosis type 2 is a dominantly inherited tumour predisposition syndrome caused by mutations in the *NF2 *gene on chromosome 22. More than 50% of patients represent new mutations and as many as one-third are mosaic for the underlying disease-causing mutation. Although truncating mutations (nonsense and frameshifts) are the most frequent germline event and cause the most severe disease, single and multiple exon deletions are common. A strategy for detection of the latter is vital for a sensitive analysis. Diagnosis is based on clinical and neuroimaging studies. Presymptomatic genetic testing is an integral part of the management of NF2 families. Prenatal diagnosis and pre-implantation genetic diagnosis is possible. The main differential diagnosis of NF2 is schwannomatosis. NF2 represents a difficult management problem with most patients facing substantial morbidity and reduced life expectancy. Surgery remains the focus of current management although watchful waiting with careful surveillance and occasionally radiation treatment have a role. Prognosis is adversely affected by early age at onset, a higher number of meningiomas and having a truncating mutation. In the future, the development of tailored drug therapies aimed at the genetic level are likely to provide huge improvements for this devastating condition.

## Disease name and synonyms

Neurofibromatosis type 2(NF2), Bilateral acoustic neurofibromatosis, Central neurofibromatosis. OMIM #101000

## Definition

Neurofibromatosis type 2 (NF2) is a dominantly inherited tumour prone disorder characterised by the development of multiple schwannomas and meninigiomas [1]. The disease can be diagnosed when a pathogenic mutation in the NF2 gene is identified or when the criteria in table 1[1] are fulfilled.

**Table 1:** Diagnostic criteria for Neurofibromatosis type 2 (these include the NIH criteria with additional criteria)

Main criteria	Additional criteria
Bilateral vestibular schwannomas (VS) *or *family history of NF2 *plus*	Unilateral VS *plus *any two of: meningioma, glioma, neurofibroma, schwannoma, and posterior subcapsular opacities
1) Unilateral VS *or*	or
2) Any two of: meningioma, glioma, neurofibroma, schwannoma, posterior subcapsular lenticular opacities	Multiple meningioma (two or more) *plus *unilateral VS *or *any two of: glioma, neurofibroma, schwannoma, and cataract

NF2 – Neurofibromatosis type 2; NIH – National Institutes of Health; VS – vestibular schwannomas

## Epidemiology

When using established clinical diagnostic criteria [1] and based on mutations in the NF2 gene [2,3] assessment of the frequency of NF2 in the population can be made. This is complicated by the high rate of mosaicism [4]. There have only been two epidemiological studies of NF2 one in North West England [5,6], [Evans DG et al. Birth incidence and prevalence of tumour prone syndromes: estimates from a UK genetic family register service. Am J Med Genet 2009. unpublished] and one in Finland [7]. The incidence of NF2 was initially reported as 1:33–40,000 individuals in a 4 million population in England [5]. Disease prevalence was somewhat lower at 1: 200,000. However, a recent update suggests that the incidence may be as high as 1:25,000 [6]. Disease prevalence has now risen to around 1 in 60,000 due to earlier diagnosis and better survival due to improved treatment [Evans DG et al. Birth incidence and prevalence of tumour prone syndromes: estimates from a UK genetic family register service. Am J Med Genet 2009. unpublished]. A lower incidence of 1 in 87,410 was reported in a 1.7 million population in Finland [7].

## Clinical description

NF2, in contrast to neurofibromatosis type 1 (NF1) is characterised by the development of schwannomas, meningiomas and ependymomas, with the great majority of patients developing bilateral schwannoma involvement of the superior vestibular branch of the eighth cranial nerve [1]. Although the disease is still classified as "neurofibromatosis", neurofibromas are relatively infrequent. The first clear description of NF2 was in 1822 by Wishart [8]. NF1 was fully delineated in the late nineteenth century by von Recklinghausen. However, it was the eminent Harvey Cushing, who described bilateral eighth nerve tumours occuring as part of von Recklinghausen disease in 1916 [9]. This description is largely responsible for the confusion between the two conditions which continued for many years. Indeed, the literature prior to 1985 has many NF2 cases being described as part of von Recklinghausen disease, with bilateral vestibular schwannomas (VS) being included in a major patient series [10].

The hallmark of NF2 is the development of bilateral VS. VS usually present with hearing loss, tinnitus or imbalance or a combination of the three symptoms. The other main tumour features are schwannomas of the other cranial, spinal and peripheral nerves; meningiomas both intracranial (including optic nerve meningiomas) and intraspinal; and some low-grade central nervous system (CNS) malignancies (ependymomas and gliomas). Four large clinical studies have now confirmed this clinical picture [1,11-13] (Table 2). Individuals may present with cranial meningiomas or a spinal tumour long before the appearance of a VS.

**Table 2:** Clinical characteristics of Neurofibromatosis type 2 patients in four studies

Characteristic	Study			
	Kanter et al. [11]	Evans et al. [1]	Parry et al. [12]	Mautner et al. [13]

Number of cases	73	120	63	48

Number of families	17	75	32	44

Sporadic cases	0	45	17	44

Mean age at onset (years)	20 (of 59)	22	20	17

Intracranial meningiomas (%)	18	45	49	58

Spinal tumours (%)	NA	26	67	90

Skin tumours (%)	32 (of 73)	68 (of 100)	67	64

Café-au-lait macules (%)	42 (of 31)	43 (of 100)	47	NA

Cataract (%)	NA	38 (of 90)	81	62

Intracranial astrocytoma (%)	NA	4.1	1.6	NA

Ependymoma (%)	NA	2.5	3.2	6

Optic sheath meningioma (%)^1^	NA	4.1	4.8	8

^1^In Mautner et al., the frequency of optic nerve sheath tumours is for all histological types (i.e., schwannomas and meningiomas).

The majority of individuals with NF2 present with hearing loss, which is usually unilateral at time of onset. The hearing loss may be accompanied or preceded by tinnitus. VS may also cause features such as dizziness or imbalance as the first symptom. Nausea, vomiting or true vertigo are rare symptoms except in late stage disease.

A significant proportion of cases (20–30%) present with symptoms from an intracranial meningioma (headaches, seizures), spinal tumour (pain, muscle weakness, paraesthesia), or cutaneous tumour [1,11-13]. Indeed, the first sign of more severe multi-tumour disease in early childhood is often a non-8^th ^nerve tumour (including a cutaneous tumour), or an ocular presentation [14]. Adult presentation is thus quite different to paediatric presentation, in which VS accounts for as little as 15–30% of initial symptoms. There also appears to be a tendency to mononeuropathy, particularly affecting the facial nerve causing a Bell's-like palsy, which does not fully recover years before the detection of a VS. Some children present with a polio-like illness with wasting of muscle groups in a lower limb, which again does not fully recover. In adulthood, a more generalised symptomtatic severe polyneuropathy occurs in about 3–5% of patients, often associated with an "onion bulb" appearance on nerve biopsy [1]. This can progress, leading to severe muscle wasting and even death. However, around 40% of patients will show evidence of polyneuropathy on nerve conduction studies [15].

Ophthalmic features are also prominent in NF2. Patients often suffer from reduced visual acuity of various causes. Between 60–80% of patients have cataracts [1,10,11], which are usually presenile posterior subcapsular lenticular opacities that rarely require removal. However, cortical wedge opacities may be present from near birth. Optic nerve meningiomas can cause visual loss in the first years of life and extensive retinal hamartomas can also affect vision. Misdiagnosis of both of these abnormalities as retinoblastoma has led to the eye being removed in the first few years of life.

The skin is a useful aid to diagnosis, but cutaneous features in NF2 are much more subtle than in NF1. About 70% of NF2 patients have skin tumours, but only 10% have more than ten skin tumours. The tumours appear to be of at least three different types. The most frequent type is a plaque-like lesion, which is intra-cutaneous, slightly raised and more pigmented than surrounding skin, often with excess hair. More deep-seated subcutaneous nodular tumours can often be felt, sometimes on major peripheral nerves. These tumours occur as a fusiform swelling of the nerve with thickened nerve palpable on either side. There are also occasional intracutaneous tumours similar to those in NF1. The great majority of these tumours are schwannomas, but occasional definite neurofibromas do occur.

## Etiopathogenesis

NF1 and NF2 were eventually recognised as separate genetic and clinical diseases with the localisation of the respective genes to chromosome 17 and 22 [16,17]. This was followed by the formal clinical delineation at a National Institutes of Health (NIH) consensus meeting in the USA in 1987 [18]. It is now clear that NF2 is a genetically homogeneous condition, with no evidence for another genetic cause of classical NF2 (bilateral VS).

Seizinger *et al*. were the first to show loss of constitutional heterozygosity of chromosome 22, with DNA markers lost in tumours from a patient with NF2 [19]. As such NF2 was one of the first inherited tumour prone disorders to be localised to a specific genetic location. Linkage studies then confirmed that all affected members of the large family carried the same copy of chromosome 22q. The *NF2 *gene was then isolated by the simultaneous discovery of constitutional and tumour deletions in a gene coding for a cell membrane-related protein, which has been termed merlin or schwannomin by the two groups who isolated it [2,3].

### Constitutional mutations

Standard mutation techniques, such as Single Strand Conformational Polymorphism (SSCP) analysis or Denaturing Gradient Gel Electrophoresis, detected between 35% and 66% of pathogenic mutations [20-23]. The majority of these mutations were truncating mutations, leading to a smaller and probably non-functional protein product. Early studies suggested that missense mutations (which result in a complete protein product) and large deletions (which result in no protein product) both caused predominantly mild phenotypes. Larger studies of detailed genotype/phenotype correlations in multiple families have confirmed this finding [21-26]. Phenotype is more variable in patients with splice-site mutations, with milder disease in patients with mutations in exons 9–15 [26,27]. Definitive evidence from effects on survival have been established with missense mutation patients having statistically greater survival than nonsense and frameshift mutations [26]. As in other tumour prone disorders the frequency of large genomic rearrangements did not become apparent until some time after the gene was cloned, due to the poor availability of methods to allow routine clinical screening for their presence. About half of these are detectable with standard cytogenetic analysis with FISH [28]. A small number of constitutional abnormalities such as chromosome translocations and ring 22 would still need a cytogenetic analysis to confirm their presence. The majority of large scale rearrangements are now routinely detected by Multiplex Ligation-dependant Probe Amplification (MLPA) and are known to account for around 15% of NF2 germline aberrations [28,29]. The commonest of this class of mutations is a deletion of the NF2 promoter, exon 1 and most of intron 1 and deletions of the whole gene, indeed combined these two account for over half of all MLPA abnormalities (Table 3). The differing frequency of the various types of NF2 mutation between familial, sporadic and mosaic cases can be seen in Table 4. This represents the output from one centre dedicated to whole gene analysis with the great majority of patients having been screened by a technique capable of the detection of large deletions or duplications. More extensive lists of NF2 mutations described worldwide are available online and in recent reviews [30-32], but these are less likely to reflect the true ratios of different classes of mutations, as a variety of different techniques have been used. The sensitivity of genetic testing using sequence analysis and MLPA can be derived from column 1 (Table 1). Ninety-three per cent of mutations have been identified in the second generation of NF2 families. Of the missing eight families six have been subjected to more detailed analysis including RNA analysis. Two putative splicing variants deep in the introns have been identified based on Protein Truncation Testing and intronic sequencing (Messaien L, personal communication), but no other mutations have been found.

**Table 3:** MLPA abnormalities in 62 unrelated families

MLPA abnormality	Number of occasions (mosaic)
Exon 1-intron1 (deletes intronic CA repeat)	18 (3)

Whole gene (exons 1–17) deletion	16 (2)

Exons 5–17 deletion	3 (2)

Exons 2–3 deletion	2

Exons 2–10 deletion	2

Exons 13–15 deletion	1

Exons 1–16 deletion	2 (1)

Exons 1–10 deletion	3 (1)

Exons 1–3 deletion	1

Exons 1–4 deletion	2 (1)

Exons 1–2 deletion	1 (1)

Exons 15–17 deletion	1

Exons 8–17 deletion	1

Exons 8–15 deletion	1

Exon 3 deletion	1

Exon 7 deletion	1

Exon 5 deletion	1

Exons 12–14 duplication	1

Exons 10–16 duplication	1

Exon 2 deletion	1

Exon 8 deletion	1

Exons 2–4 deletion	1

MLPA- Multiplex Ligation-dependant Probe Amplification

**Table 4:** Mutations identified in 529 families with Neurofibromatosis type 2 in the Manchester (UK) genetics laboratory

Type of mutation	Detection in 2^nd ^generation (n = 108)	Detection in sporadic non mosaic patients (% non mosaic)	Mosaic mutations (% of mosaic)	Total
Splice site	35 (32%)	43 (22%)	3 (4%)	80 (15%)

MLPA positive	23 (20%)	28 (14%)	11 (15%)	62 (12%)

FSD	18 (17%)	36 (18%)	20 (26%)	74 (13%)

Nonsense	16 (15%)	67 (35%)	28 (37%)	111 (22%)

Missense	7 (6%)	5 (3%)	1 (1%)	13 (2.5%)

FSI	3 (3%)	11 (7%)	6 (8%)	20 (4%)

IFD	1 (1%)	1	3 (4%)	5 (1%)

Ring 22	0	0	3 (4%)	3

Not found	7 (7%)	230 (55%)	158	166/529 (31%)

Total	108	191/421 (45%)	72	529

A considerable proportion of NF2 patients, particularly milder cases, have mosaic disease, in which only a proportion of cells contain the mutated *NF2 *gene. The initiating mutation occurs after conception, leading to two separate cell lineages. The proportion of cells affected depends on how early in development the mutation occurs. Recent evidence suggests that up to 20–30% of NF2 cases without a family history of the disease are mosaic, carrying the mutation in too small a proportion or none of their lymphocytes to be detected from a blood sample [4,33-35]. This accounts for the milder disease course in many individuals with unfound mutations, and since only a subset of germ cells (or none) will carry the mutation, there is less than a 50% risk of transmitting the disease to their offspring. However, if an offspring has inherited the mutation, they will have a typical phenotype and usually be more severely affected than their parent, since the offspring will carry the mutation in all of their cells. One of the features that suggested that mosaicism existed in NF2 was that *NF2 *mutations were harder to find in blood in isolated non inherited cases than in patients who had inherited the disease from an affected parent. Mosaicism may be particularly likely in NF2 if the tumours are predominantly on one side of the body. The mosaic mutation can be detected by analysing tumour material from an affected individual. If an identical mutation is found in two tumours from that individual, their offspring can be tested for the presence of the mutation. Also when two alterations, either: two mutations or one mutation plus one allele loss, are found in on tumour, one of these must be the constitutional one. Thus offspring can be tested for both abnormalities and potentially exclude inheriting NF2.

C > T transitions leading to a nonsense mutation are the most common mutations in the *NF2 *gene [30-32]. These account for the only significant variation in mutation frequency across the NF2 gene apart from the lack of mutations in exons 16 and 17 and a relatively low frequency in exon 9 [30].

### Somatic mutations

There are notable similarities, but also differences between the distribution and type of germline and somatic *NF2 *mutations. Among germline mutations in classic NF2, nonsense mutations are more common than frameshift mutations by a ratio of 1.3:1, but this ratio is reversed for somatic mutations [36]. In both situations *NF2 *nonsense mutations, C > T transitions in CGA codons and non-CGA codons are the most common single base-pair transitions. However there is a marked absence of mutations in exons 14 and 15 in sporadic meningioma. One recently reported phenomenon is the increasing drift from nonsense mutations in the germline and early somatic mutations to a predominance of frameshift mutations in the tumours of older patients with vestibular schwannoma [36]. This is likely to be due to a deficiency in certain DNA repair pathways in older patients [36].

Although it is thought that effectively all schwannomas require inactivation of the NF2 gene, no reports apart from those studying NF2 protein have confirmed a 100% knock out of both copies of the NF2 gene. This is likely to be due to diversity of mechanisms that inactivate the gene. The standard approach of mutational analysis and Loss of Constitutional Heterozygosity (LOH) on tumours will detect involvement of NF2 in about 80–90% of schwannomas, but both copies can only be confirmed as affected in about 50–60% of cases. The mechanism of LOH is also not straightforward, whilst most cases involve loss of chromosome 22 or at least the long arm, a proportion are now known to be due to mitotic recombination with essentially two identical copies of a mutated NF2 gene and distal 22q [37]. This mechanism is now thought to be a primary cause of NF2 inactivation in schwannomatosis. It is now known that between 20–40% of sporadic schwannomas are inactivated by NF2 methylation [38,39] and in a similar fashion to *TP16 *this could involve both copies of NF2 and explain why some tumours do not harbour identifiable point mutations or LOH. Approximately 60% of sporadic meningiomas have NF2 gene involvement and promoter methylation again plays a significant role [40].

## Diagnosis

The Manchester (modified NIH) diagnostic criteria for NF2 are shown in Table 1. The original NIH criteria have been expanded to include patients with no family history who have multiple schwannomas and or meningiomas, but who have not yet developed bilateral 8^th ^nerve tumours. Patients who have asymmetric involvement are likely to be mosaic [41]. At very young ages (< 18 years) individuals presenting with an apparently isolated meningioma [14] or vestibular schwannoma [42] are have a 20% and 10% likelihood respectively of developing NF2. However after 20 years of age this rate drops dramatically and the diagnosis becomes very unlikely after 30 years of age [42].

### Diagnostic methods

• Clinical and family history

• Examination including cutaneous and ophthalmic (Slit lamp)

• Craniospinal MRI

• Molecular analysis

## Differential diagnosis

The main differential diagnosis of NF2 is schwannomatosis and some patients with multiple non cranial schwannomas turn out to have mosaic NF2 [34,35]. However, patients fulfilling the most sensitive Manchester criteria are unlikely to be misclassified [43].

## Antenatal diagnosis and genetic counselling

NF2 is an autosomal dominant disease with usually a 50% risk of transmission from an affected individual to their offspring. This was first confirmed in a large family reported by Gardner and Frazier in 1930. 50–60% of patients have no family history and represent *de novo *mutations in the NF2 gene [1-3]. Individuals who inherit a pathogenic mutation in the *NF2 *gene will almost always develop symptoms by 60 years of age [7]. Exceptionally, patients particularly in the pre MRI era will have not been diagnosed in their lifetime. Although the transmission rate is 50% in the second generation and beyond, the risk of transmission in an apparently isolated patient with NF2 is less than 50% due to mosaicism [4].

Because detection of tumours at an early stage is effective in improving the clinical management of NF2, pre-symptomatic genetic testing is an integral part of the management of NF2 families. Once a mutation has been identified in an affected individual, a 100% specific test is available for the family. However, mutation screening may not reveal the causative mutation. Predictive diagnosis by linkage analysis using intragenic markers or markers flanking the *NF2 *gene is also possible in the great majority of families with two or more living affected individuals. In the absence of a genetic test a cumulative age at onset curve [44] can be used. Age at onset curves aid genetic counselling; for example, the risk of having inherited NF2 for an asymptomatic at-risk individual 25 years of age, prior to screening, drops to 25%. The risk to an unaffected 30 year old with a normal scan would be < 10%. Tumour analysis plays a vital role in providing genetic testing for the offspring of sporadic patients. Indeed analysis should if possible first be carried out on tumour so that a targeted approach can be used on the blood sample. If both mutational events are identified in the NF2 gene in a tumour and neither is present in the blood the patient must be mosaic for one of these mutations [33-35,45,46]. Even if only LOH is identified this still allows exclusion of NF2 in 50% of offspring if they can be shown to have inherited the allele "lost" in the tumour [35,46]. At-risk individuals who are shown not to have inherited the mutated *NF2 *gene do not need further follow-up.

Even in the absence of identifying a mutation the residual risk of NF2 can be substantially reduced in the child of an apparently isolated case. In particular patients presenting with asymmetric disease over 40 years of age with negative mutation analysis in blood would have a very low chance of transmitting NF2 to their children (Table 5).

**Table 5:** Transmission risks to offspring for isolated cases of Neurofibromatosis type 2 before and after negative mutation testing.

	Number	PRE testing Mosaic inferred	PRE testing Transmission risk	POST genetic negative testing in blood Mosaic inferred	POST genetic negative testing in blood Transmission risk
< 20 BVS	85	12%	45%	46%	30% 1 in 3

< 20 UVS	21	42%	33%	87%	11% 1 in 9

20–29 BVS	67	27%	36%	78%	16% 1 in 6

20–29 UVS	27	78%	19%	97%	8% 1 in 12

30–39 BVS	54	50%	28%	88%	11% 1 in 9

30–39 UVS	19	85%	12%	98%	6% 1 in 16

40+ BVS	53	63%	22%	93%	9% 1 in 11

40+ UVS	34	90%	10%	99%	5% 1 in 20

Results are based of outcomes of testing in first affected family members and on age at onset and laterality of presentation with vestibular schwannomas (VS).

BVS – presentation with bilateral VS; UVS – initial presentation with unilateral VS

Because of the severity of NF2 there is a demand for prenatal diagnosis and pre-implantation genetic diagnosis. Use of the techniques above means that this is possible in the great majority of families.

### Screening protocol

Children of affected patients should be considered to be at 50% risk of NF2 and screening for NF2 can start at birth. Cataracts can affect vision in early life and other tumour implications are present in the first ten years of life, particularly cranial meningiomas. Formal screening for VS should start at ten years, as it is rare for tumours to become symptomatic before that time even in severely affected families. Annual audiological tests including auditory brainstem response are still a useful adjunct to MRI [44]. Surgery is unlikely to be more successful for tumours < 6 mm than for tumours sized 6 mm, but VS growth is higher in younger patients, so for asymptomatic at-risk individuals without tumours, MRI screening every two years for those < 20 years old and every 3–5 years for those age > 20 years should be sufficient. The initial MRI scan could be at around 12 years of age, or 10 years in severely affected families. Once tumours are present, MRI screening should probably be at least annual. Spinal tumours are seen in 60–80% of NF2 patients on MRI [47-50]. While only 25–30% of patients with spinal tumours require a spinal operation from a symptomatic tumour, a full annual neurological examination is probably a wise precaution with Spinal MRI only every 3 years or if there are new symptoms. If no tumours are present on the initial scan a further scan 5–10 years later may be reasonable.

In most families it is now possible to develop a genetic test so that screening can be targeted to affected individuals only. Identifying the affected patient's mutation not only allows testing of at risk relatives, but may also give important indicators as to the patient's own prognosis. As 25–30% of NF2 patients are mosaic frozen tumour should be taken at operation (with patient consent) for genetic tests.

## NF2 management

NF2 presents many difficult management dilemmas. The mainstay of management of NF2 is surgical removal of symptomatic cranial and spinal tumours. The timing of removal of vestibular schwannomas is a more difficult area. Surgical results are certainly far better when managed by an experienced team [50-52]. There is clear evidence of a reduction in mortality with a significantly increased life expectancy for NF2 patients managed at 3 specialty centres in the UK (OR 0.34) [53]. It is important to balance the use of microsurgery and radiation treatment, which can have a role in patients who have particularly aggressive tumours, or who are poor surgical risks, or who refuse surgery. Teams experienced in the positioning of brainstem implants can offer partial auditory rehabilitation to those who are deaf, although results are still behind those achievable for cochlear implants. Although the cochlear nerve may be left initially intact after surgery its blood supply may be damaged, nonetheless a few patients can be rehabilitated successfully with a cochlear implant.

### Outcomes

Even with improvements in microsurgery and with use of radiation therapy, the great majority of individuals with NF2 become completely deaf. The tumours in NF2 are more difficult to treat than those of sporadic unilateral VS, as NF2 VS are often multifocal, appearing "like a bunch of grapes" around the vestibular nerve in particular. There is evidence for a histological difference, with NF2 VS being more lobular and less vascular then their sporadic counterparts [54]. This leads to a greater risk of facial nerve damage in NF2. Loss of facial nerve function is one of the most feared aspects of the condition for many sufferers, although in good surgical hands this complication is now much less common [50,52]. Patients may also be severely disabled by a combination of poor balance, visual problems and weakness due to spinal tumours. Indeed, many NF2 patients become wheelchair-bound in early adulthood. Many patients with multi-tumour disease die in their twenties and thirties. In view of the multiplicity of problems affecting many patients it is strongly recommended that NF2 patients are managed by a multidisciplinary team in specialist centres [52].

## Specialty centres

A typical NF2 specialist centre will require involvement of a number of key staff members.

### Permanent clinic staff

Neurosurgeon, Otolaryngologist, physician (neurologist/geneticist), clinic nurse/patient link worker and a dedicated clinic secretary.

Clinic equipment: lightwriter/pallantype/voice activated software.

In order to minimise patient visits morning scanning with lunchtime radiological review and an afternoon clinic is preferable.

### Each clinic should have access to a named

Neuroradiologist, ophthalmologist, peripheral nerve surgeon, plastic surgeon, neurologist/geneticist (if not involved in main clinic), hearing therapist, physiotherapist, paediatrician.

Patients should be given the option of a radiation therapy opinion, which will only be available at a few centres.

### Managing affected children

NF2 is being recognised more and more frequently in childhood often before VS have developed. Recognition of the more severe disease course with early presentation and the more atypical features such as mononeuropathy are important.

### Surgery

VS in NF2 are more difficult to treat than those of sporadic unilateral VS because NF2-related VS are often multifocal in the eighth nerve complex, and the potential for associated facial nerve schwannomas. Surgery to remove VS in NF2 almost always leads to total deafness with loss of the cochlear nerve. Despite the great improvement in VS surgery over the last three decades, facial nerve damage and other adverse outcomes remain a real possibility during tumour removal, especially in the hands of less experienced VS surgeons. Facial weakness may threaten the health of the eye as a result of loss of the protective blink reflex. and as the lacrimal gland is also supplied by the facial nerve the loss of tear production will increase this risk. If facial nerve damage coexists with loss of corneal sensation from damage to the trigeminal nerve then the eye becomes exceptionally vulnerable to corneal ulceration and blindness.

The cornerstone of modern NF2 management is conservation of function, and the maintenance of "quality of life". The mere presence of a tumour is not an indication for its removal. Serious thought must be given to the benefits that are sought and the risks and complications of the surgery, and the treatment must be tailored to the needs of the individual patient [52]. Attempts at hearing preservation surgery should be limited to experienced centres who can offer a realistic chance of maintaining both the cochlear nerve, but also cochlea function. If hearing is lost after apparent cochlear nerve preservation the patient may still be suitable for a cochlear implant. In many if not most instances the best policy will be to observe VS to decide on the best time to balance surgical morbidity against the almost inevitable loss of hearing.

The principle of minimal interference for VS applies equally to schwannomas on other cranial nerves, to intracranial meningiomas, and to spinal tumours. It is very uncommon to have to remove a schwannoma growing on a cranial nerve other than the eighth because these tumours appear to have a much slower growth pattern than NF2 VS. Spinal tumours are mostly considered for excision if they are clearly producing symptoms or physical signs. In the absence of any dramatic growth of tumours, the head should be scanned every year and the spine every 3 years.

## Radiation therapy

It is important to balance the use of microsurgery and radiation treatment, which has a role in patients who are poor surgical candidates, or who refuse or wish to avoid surgery because of its associated risks. Radiation therapy should be *mentioned *as a management option, even if the tumour is larger than the size criterion for treatment. The upper limit of size for radiotherapy is generally a maximum intracranial diameter of 3 cm [55]. The patient should be aware of the management options even if the tumour is not suitable for a particular treatment modality. In NF2 cases selected for radiosurgery tumour control rates are of the order of 50%, with 40% retaining pre-treatment hearing for at least 3 years [56]. This is nonetheless substantially worse than for sporadic vestibular schwannomas.

Surgeons should use clinical judgement as to when to recommend radiation therapy [52]. Follow-up for life with interval scanning is necessary, although this would be required for NF2 anyway. Patients should be made aware of the variable reported outcomes of the treatment and the risk of the radiation-induced malignant change, which has been reported disproportionately more in NF2 than sporadic patients [57,58]. The tumour may also be more difficult to excise after radiotherapy, and that reported facial nerve outcomes after surgery following stereotactic radiation therapy are frequently poor [59].

### New therapies

The NF2 protein appears to impact on multiple intracellular signalling pathways. These pathways include the PI3-kinase/Akt, Raf/MEK/ERK and mTOR pathways [60]. In particular, studies using NF2-derived tumour tissue reveal elevated levels of phosphorylated Akt. More recently, Akt-dependent phosphorylation of Merlin on 2 residues (Thr-230 and Ser-315) has been shown to target Merlin for ubiquitin-dependent degradation. Although no PI3 kinase or Akt inhibitors have yet been approved for treatment, there are multiple compounds in development – primarily for oncology indications. The Raf/MEK/ERK pathway has been implicated in NF2 tumorigenesis in part through identification of elevated levels of phospho-ERK and phospho-MEK.

The progress being made in cellular research especially with regard to pathways in which the NF2 gene product interacts raises the hopes of targeted therapy. Targeting the ERK1/, AKT, integrin/focal adhesion kinase/Src/Ras signaling cascades, PDGFRbeta, phosphatidylinositol 3-kinase/protein kinase C/Src/c-Raf pathway, VEG-F and other pathways [60,61] means that drugs such as avastin, elotinib [62], lapatinib and sorafenib [63] may well bear fruit.

## Prognosis

NF2 remains a life limiting and life spoiling condition. Patients diagnosed prior to 1990 had only a 15 year life expectancy from diagnosis [1,64]. Improvement in management and early diagnosis are improving these outcomes but many people with NF2 still die very young. Prognosis is adversely affected by early age at onset, number of meningiomas and having a truncating mutation [52].

## Unresolved questions

1. What is the most appropriate molecular/cell biological target(s) for therapy

2. What are the long term safety issues of radiation treatments and does fractionated radiotherapy offer advantages over sterotactic or gamma knife treatment

## Conclusion

NF2 remains a condition that is life spoiling and life limiting. Multidisciplinary management with early diagnosis are mainstays of management. Hopefully new targeted therapies will revolutionise the outcomes in this condition.

## Abbreviations

NF2: neurofibromatosis type 2; MRI: magnetic resonance imaging; NF1: neurofibromatosis type 1; VS: vestibular schwannomas; CNS: central nervous system; NIH: National Institutes of Health; SSCP: single strand conformational polymorphism; MLPA: multiplex ligation-dependant probe amplification; LOH: loss of constitutional heterozygosity.

## Competing interests

The author declares that they have no competing interests.
